# Flipped classroom improves student learning in health professions education: a meta-analysis

**DOI:** 10.1186/s12909-018-1144-z

**Published:** 2018-03-15

**Authors:** Khe Foon HEW, Chung Kwan LO

**Affiliations:** The University of Hong Kong, Pok Fu Lam, Road, Hong Kong

**Keywords:** Flipped classroom, Flipped learning, Health professions education, Meta-analysis

## Abstract

**Background:**

The use of flipped classroom approach has become increasingly popular in health professions education. However, no meta-analysis has been published that specifically examines the effect of flipped classroom versus traditional classroom on student learning. This study examined the findings of comparative articles through a meta-analysis in order to summarize the overall effects of teaching with the flipped classroom approach. We focused specifically on a set of flipped classroom studies in which pre-recorded videos were provided before face-to-face class meetings. These comparative articles focused on health care professionals including medical students, residents, doctors, nurses, or learners in other health care professions and disciplines (e.g., dental, pharmacy, environmental or occupational health).

**Method:**

Using predefined study eligibility criteria, seven electronic databases were searched in mid-April 2017 for relevant articles. Methodological quality was graded using the Medical Education Research Study Quality Instrument (MERSQI). Effect sizes, heterogeneity estimates, analysis of possible moderators, and publication bias were computed using the Comprehensive Meta-Analysis software.

**Results:**

A meta-analysis of 28 eligible comparative studies (between-subject design) showed an overall significant effect in favor of flipped classrooms over traditional classrooms for health professions education (standardized mean difference, SMD = 0.33, 95% confidence interval, CI = 0.21–0.46, *p* < 0.001), with no evidence of publication bias. In addition, the flipped classroom approach was more effective when instructors used quizzes at the start of each in-class session. More respondents reported they preferred flipped to traditional classrooms.

**Conclusions:**

Current evidence suggests that the flipped classroom approach in health professions education yields a significant improvement in student learning compared with traditional teaching methods.

## Background

Many classes in higher education institutes now employ blended learning; whereby students learn in part at a supervised face-to-face location on campus, and in part through the Internet with some elements of student choice over place and pace [1].Of the many different models of blended learning in practice, the use of flipped classroom approach has become increasingly widespread [2–4].

Initially popularized in the United States [5], flipped classrooms replace teacher-led in-class instructions with individual homework or group activities [6]. Recently, the flipped classroom approach has made inroads into health professions education, and has even been touted “a new paradigm” in medical education [7]. Various health professions have adopted this instructional approach into their curricula [8]. A recent review of learner perceptions of flipped classrooms in health professions education [8] found an overwhelming positive response from students who attended flipped courses. More specifically, students expressed high levels of satisfaction with pre-class video lectures because the videos can be accessed at any time and as often as they desire. Students also highly regarded the use of small group discussion-based activities in flipped classroom face-to-face sessions because these sessions help increase their motivation to learn, enhance their level of engagement, and interest in the subject matter [8].

But does using the flipped classroom approach in health professions education really improve student learning? It is important to note that positive student perception toward flipped classrooms does not necessarily imply that this instructional approach will significantly improve student learning [8]. For example, flipping the evidence-based medicine (EBM) course by Ilic et al. [9] did not improve scores on the Berlin objective assessment of EBM competencies compared to its traditional counterpart, despite students reporting a positive perception of the flipped course.

Up to now the effectiveness of flipped classroom approach compared with traditional learning has not been established. Although several literature review studies have been conducted in health care professionals such as nursing [10, 11] and medical education [8, 12], no meta-analysis has been published that specifically examines the effect of flipped classroom versus traditional classroom on student learning. By traditional classroom, we refer to the approach of having students come to class during which teachers use a range of pedagogical strategies (e.g., lecture, case discussion, student presentation), and then students complete most of their homework after school [13, 14]. The uncertainty about the effectiveness of flipped classroom approach over traditional instruction provided the impetus for the current study. We opted to contrast flipped classrooms with traditional classrooms because the latter is still widely used in health professions education [15].

### Conceptual framework

For the purposes of this study, we adopted the conceptual framework of flipped classroom approach by educause [16], one of the leading associations that focus on instructional technology in higher education, as a pedagogical strategy in which the typical lecture and homework elements of a course are reversed. In a typical traditional classroom, students listen to lectures in class and complete most of their homework after class. In a flipped classroom, students listen or watch pre-recorded lectures before class and perform active learning activities such as exercises, projects, or discussions [16]. Problem-based learning may be one of the activities used in flipped classroom [17].

Although pre-recorded lectures could be a podcast or other audio format, the use of videos has become so ubiquitous that the flipped classroom approach has come to be identified with pre-recorded videos [16, 18, 23]. Therefore, in the present review, we focused specifically on flipped classroom studies in which pre-recorded videos were provided (rather than live lectures, or intelligent tutoring system without a video or instructor) prior to face-to-face class meetings.

It is important to stress that this definition excludes the *sole* use of pre-class reading materials as a form of flipped learning. Text-based materials cannot “closely mimic what students in a traditional setting would experience” [19] since it does not involve instructors’ explanation and elaboration of contents [20]. Muir and Geiger [21] reported that “a book doesn’t really walk through the steps on how to do something.” In contrast, the use of video lectures enables the instructors to elaborate the course contents as in a traditional lecture [20, 21].

## Methods

### Data sources and search strategies

This meta-analysis and review were carried out according to the PRISMA (Preferred Reporting Items for Systematic reviews and Meta-Analysis) guidelines [22]. Relevant online databases were searched from January 2012 through March 2017. January 2012 onwards was chosen because 2012 was the year of the first publication of an application of flipped classroom approach to health professions student teaching [23]. Altogether, seven electronic databases were searched in mid-April 2017, including Academic Search Complete, PubMed, PsycINFO, CINAHL Plus, TOC Premier, British Nursing Index, and ERIC. To capture a broader range of potentially eligible articles, we employed the following search terms with Boolean operators: “(flip* or invert*) and (class* or learn* or instruction* or course*) and (medic* or nurs* or pharmac* or physiotherap* or dental or dentist* or chiropract*)”. The asterisk was used as a wild card to include most of the common expressions of the flipped classroom approach (e.g., flipped learning, flipped class, flipping the classroom). The search term was entered as a string and searched in each of the seven databases.

### Eligibility criteria

To examine the possible effects of flipped classroom on student achievement, a meta-analysis was done on eligible articles. The eligibility criteria are as follows:

#### Inclusion

- The studies must report at least one comparison of a flipped classroom condition versus a traditional classroom condition (i.e., between-subject design) focusing on health care professionals including medical students, residents, doctors, nurses, or learners in other health care professions and disciplines (e.g., dental, pharmacy, environmental or occupational health). The flipped classroom implementation *must* consist of both pre-class and in-class activities.
- The pre-class flipped classroom activities must at least include the use of instructor-recorded classroom lectures, PowerPoints with instructor talking head, YouTube videos, Khan Academy videos, TED (technology, entertainment, design) video talks, screencast, or PowerPoints with instructor’s voice over.
- In the present review, we include only flipped classroom studies that have a face-to-face meeting because face-to-face class meeting is typically used in many flipped classroom implementations [18, 24]. Comparing online courses (without face-to-face meeting) with flipped classrooms is outside the scope of our review.
- The traditional classroom involves students coming to class during which teachers typically give a lecture [25], and might use other presentation strategies (e.g., case discussion, group-work, student presentation).
- Only comparative studies such as randomized controlled trial, quasi-experiments, and historical cohort controlled research designs were included for review.
- Articles must measure student learning on similar course topics using some form of objective-based assessments such as post-tests or exams. These assessment instruments must be similar or identical.
- No geographical restrictions were imposed, however the articles must be written in English and published in peer-reviewed journals. Searching for peer-reviewed publications is a useful criterion for selecting studies of sufficient quality [26].

#### Exclusion

a) Published studies were excluded if their datasets or results were incomplete – such as if they lacked sufficient information to calculate effect sizes (e.g., sample sizes), or if effort to obtain data from corresponding authors was not successful.

### Study selection and data extraction

The title and abstract of the studies from initial the search process were screened in order to derive a preliminary set of full articles for potential final review. This was followed by a reading of the preliminary full articles by the authors individually to confirm the relevance of the studies before a final decision (through discussion) was made to confirm the studies to be included in the final review. To extract the data, we looked for information including authors of the study, publication year, location in which the study was conducted, subject topic, participant sample, study design such as quasi-experiments, randomized control trials, or historical controls, and details of the flipped classroom implementation such as the types of pre-class and in-class activities used. The percent agreement between the coders concerning the data extraction was high – 95%. To reach consensus, the discrepancies between the extracted data of the two researchers were reviewed, discussed, and resolved prior to data entry and analysis.

For all the eligible studies, one effect size was calculated for each study to meet the assumption of the independence of the effect sizes based on independent samples of students. In cases where articles reported multiple assessments of a single course subject, we selected the assessment that was most summative, as suggested by Freeman et al. [27]. For example, we chose final exam over other assessments (e.g., mid-term exam, weekly quiz). We also chose assessment that is recognized as a widely-used inventory [27] – for example, the Objective Structured Clinical Examination over an instructor-written examination. In cases, where a single study had multiple outcomes from different course subjects from the same set of students, we computed a single combined effect size using the formulas proposed by Borenstein et al. [28]. In doing so, we assumed that the correlation between the different outcomes within a comparison was 1 as suggested by Freeman et al. [27] since the same students were sampled for each outcome. This is a conservative measure as the actual correlation between outcomes is likely lower than 1 [27].

### Meta analyses

We computed effect sizes using the Comprehensive Meta-Analysis Version 3 software (Biostat, Inc., Englewood Cliffs, NJ, USA). All reported *p* values are two-tailed, unless otherwise reported. To compare the effect sizes, we used a random effects model, or random effects analysis because conditions that could affect student achievements differed among studies in the analysis, including the frequency of lessons flipped, student population, and course level. We computed effect sizes using standardized mean differences (SMDs) from the means and standard deviations of student achievement data (e.g., exam scores, post-test scores). If standard errors were used in the previous empirical studies but not the standard deviations, we used the following formula [29] to calculate the standard deviations:

$$ SE=\frac{SD}{\sqrt{\mathrm{sample}\ \mathrm{size}}}. $$

If the means and standard deviations were not reported in the previous empirical studies, the standardized mean difference was estimated using a variety of sources, including *t-*tests (or formulas, see Borenstein et al. [28]; Lipsey & Wilson [30]). The presence of heterogeneity (i.e. the degree of inconsistency in the studies’ results) was detected by the *I*^2^ test. Publication bias (or otherwise known as file-drawer problem) occurs when researchers publish only favorable results [63]. Therefore, in order to determine whether the present review suffers from publication bias, we conducted the following standard tests used to analyze publication bias including: (a) assessing the funnel plot, (b) computing Begg and Mazumdar rank correlation, (c) calculating Egger’s regression, (d) computing Duval and Tweedie’s trim and fill, and (e) calculating the classic fail-safe *N* test. Currently, sufficient information was available for all these tests.

We also conducted subgroup analyses across six major categorical moderator variables in order to identify the possible source of variation among the effect sizes and the differences among the subgroups. These moderator variables were: (a) student initial equivalence, (b) instructor equivalence, (c) research design, (d) types of students; (e) pre-class component of flipped classroom, and (f) in-class component of flipped classroom.

To determine student initial equivalence, we examined whether the study design was based on the following categories: (a) comparative studies where authors provided no data or no statistical control on student equivalence in terms of initial academic performance, or where authors merely claimed students were equivalent but did not provide any relevant statistical evidence (e.g., t-test results); or (b) comparative studies where data indicated no statistical difference on a pretest that directly relate to the topic, or on a metric of academic performance (e.g., college GPA). To evaluate instructor equivalence, we checked whether the study involved: (a) identical instructor for the flipped-and-non-flipped classes; or (b) different instructors for different classes; or (c) no data provided.

In addition, we stratified the analysis according to research design (randomized control, quasi-experiment, or historical control), types of students (e.g., medicine, pharmacy, public health, etc.), pre-class component (availability of pre-class assessment/exercise or not, availability of readings/notes or not), and in-class component (use of quiz at start of lesson or not).

## Results

**Fig. 1:**
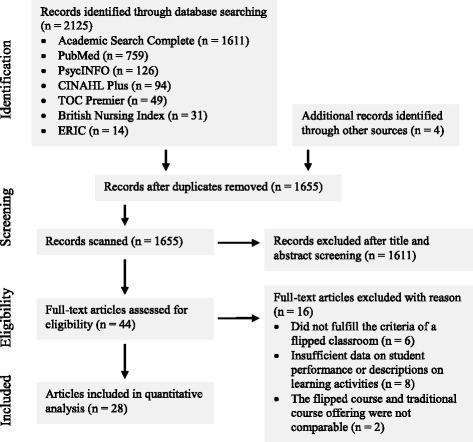
PRISMA flow diagram of article selection

The literature search process is depicted in Fig. 1. Our initial search yielded a total of 2129 articles, which was reduced to 1655 after duplicates were removed. However, after reviewing their titles and abstracts, many articles (*n* = 1611) were found to be irrelevant, particularly those that did not report empirical research, or were not related to health professions education. For example, many irrelevant search outcomes came from articles that did not collect and analyze any form of data (i.e., non-empirical), and from basic science disciplines (e.g., inverted structural plasticity, invertebrates, and tissue classification). A total of 44 full text articles were read and assessed for eligibility. Out of these 44 articles, six were removed because it did not fulfill our criteria of a flipped classroom approach, eight were removed due to insufficient data on student performance or descriptions of the learning activities, and three were removed because the flipped course and traditional course offering were not comparable. Twenty-eight articles were included in the final analysis.

### Study demographics

Table 1 descriptively summarizes the main elements of the 28 studies included in the meta-analysis. Most of the studies were based on historical control designs. Only four studies were quasi-experiments. The remaining four were randomized controlled trials.

**Table 1:** Overview of studies included in the meta-analysis

Study	Country of origin	Subject matter	Sample	Study design	Duration of intervention
Bakr et al. (2016) [31]	Australia	Dental anatomy	1st year dental students	Historical control	13 weeks
Bossaer et al. (2016) [32]	USA	Pharmacy	3rd year pharmacy students	Historical control	1 semester (actual duration not specified)
Cheng et al. (2016) [33]	China	Histology	1st year medicine students	Quasi-experiment	1 semester (actual duration not specified)
Cotta et al. (2016) [13]	USA	Pharmaceutical calculations	1st year pharmacy students	Historical control	10 weeks
Galway et al. (2014) [34]	Canada	Environmental and occupational health	1st or 2nd year graduate public health students	Historical control	13 week
Gillispie (2016) [35]	USA	Obstetrics and gynecology	3rd/4th year medical students	Historical control	8 weeks^a^
Giuliano and Moser (2016) [36]	USA	Drug literature	1st year pharmacy students	Historical control	1 semester (actual duration not specified)
Harrington et al. (2015) [37]	USA	Nursing	Baccalaureate students	Randomized	4 months
Hsu et al. (2016) [38]	Taiwan	General medicine	Postgraduate year medical students	Historical control	12 months
Ilic et al. (2015) [9]	Australia	Evidence-based medicine	3rd year medical students	Randomized	10 two-hour teaching sessions
Kiviniemi (2014) [39]	USA	Public health	Graduate students	Historical control	1 semester (actual duration not specified)
Koo et al. (2016) [40]	USA	Pharmacotherapy	2nd year doctor of pharmacy students	Historical control	8 sessions
Liebert et al. (2016) [41]	USA	Surgery	3rd year medical student	Historical control	8 weeks
Lin et al. (2017) [14]	China	Ophthalmology	Medical undergraduates	Randomized	1 semester (actual duration not specified)
McLaughlin et al. (2013) [42]	USA	Pharmaceutics	1st year pharmacy students	Historical control	13 weeks
McLaughlin et al. (2014) [43]	USA	Pharmaceutics	1st year professional students	Historical control	13 weeks
Morton and Colbert-Getz (2016) [44]	USA	Anatomy	1st year medical students	Historical control	17 weeks
Munson and Pierce (2015) [45]	USA	Pharmacogenomics	Mixture of students with bachelor degree and students without first degree	Historical control	15 weeks
O’Connor et al. (2016) [15]	USA	Radiology	3rd or 4th year medical students	Quasi-experiment	2 weeks
Pierce and Fox (2012) [6]	USA	Pharmacotherapy	Pharmacy students	Historical control	8 weeks
Porcaro et al. (2016) [46]	Australia	Hematology	Mixture of undergraduate and postgraduate students in medical science	Historical control	1 semester (actual duration not specified)
Prescott et al. (2016) [47]	USA	Patient assessment	1st year pharmacy students	Historical control	2 semesters (actual duration not specified)
Rui et al. (2017) [48]	China	Medical diagnostics	Junior-year medical students	Randomized	3 weeks
Sajid et al. (2016) [49]	Saudi Arabia	Hematology	3rd year medical students	Historical control	5 lectures
Street et al. (2015) [17]	USA	Physiology	1st year medical students	Quasi-experiment	6 weeks
Tune et al. (2013) [50]	USA	Physiology	1st year graduate medical students	Quasi-experiment	1 semester (actual duration not specified)
Whillier and Lystad (2015) [51]	Australia	Neuroanatomy	2nd year undergraduate chiropractic students	Historical control	5 weeks
Wong et al. (2014) [52]	USA	Pharmacy	1st year pharmacy students	Historical control	1 week

^a^note: Gillispie (2016) [35] - 8 weeks information obtained from the website: https://medicine-program.uq.edu.au/current-students/placements/core-rotations

Methodological quality was graded using the Medical Education Research Study Quality Instrument (MERSQI) [53]. We summarize the study quality in Table 2. The mean MERSQI score was 12.5 on an 18-point scale.

**Table 2:** Quality of studies (*N* = 28) based on MERSQI

Scale item (max. points)	Subscale (points if present)	No. (%) present
Study design (max. 3)	Non-randomized 2-group (2)	24 (86)
	Randomized 2-group (3)	4 (14)
Sampling: no. of institutions (max. 1.5)	1(0.5)	26 (93)
	2 (1)	1 (3.5)
	3 (1.5)	1 (3.5)
^a^Sampling: response rate (max. 1.5)	< 50% or not reported (0.5)	5 (17)
	50%–74% (1) (1)	1 (3.5)
	≥75% (1.5)	22 (79)
Type of data: outcome assessment (max. 3)	Objective (3)	28 (100)
Validity evidence (max. 3)	Content (1)	28 (100)
Data analysis: appropriate (max. 1)	Appropriate (1)	28 (100)
Data analysis: sophistication (max. 2)	Beyond descriptive analysis (2)	28 (100)
Highest outcome type (max. 3)	Knowledge, skills (1.5)	28 (100)

^a^Note: Sampling (response rate) refers to the proportion of students enrolled who completed the flipped classroom approach

### Meta-analysis

**Fig. 2:**
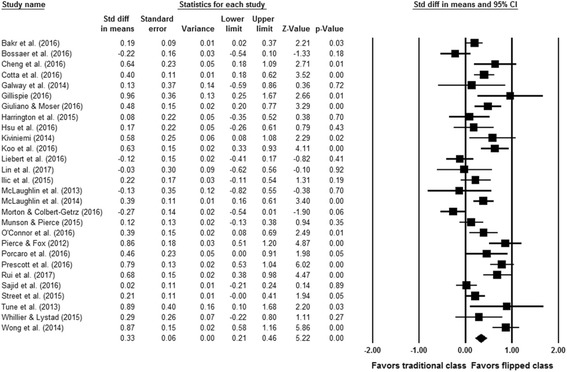
Forest plot of effect sizes (standardized mean difference) using random effect model. Note that data in Pierce and Fox [6] were provided by the corresponding author

A meta-analysis of 28 eligible comparative studies involving 2295 subjects exposed to flipped classroom and 2420 subjects exposed to traditional classroom showed an overall significant effect in favor of the flipped classroom approach for health professions education (SMD = 0.33, 95% CI 0.21–0.46, *p* < 0.001) as shown in Fig. 2. A significant *Q* statistic (p < 0.001) indicated the presence of heterogeneity (*I*^2^ = 75.6%).

### Moderator analyses

In order to explore the possible causes for the significant heterogeneity, we conducted several moderator analyses using the random-effects mode. The results of the analyses are summarized in Table 2.

When we analyzed the data based on whether the studies controlled for student or instructor equivalence, we found no evidence of heterogeneity between studies that reported initial student equivalence, studies that employed randomized student assignment, and studies that did not provide any such data (*Q* = 0.43, df = 2, *p* = 0.81).

Analyzing variation with respect to instructor equivalence also suggested no evidence of heterogeneity (*Q* = 4.72, df = 2, *p* = 0.09). Thus, the overall effect size for student performance data appears to be robust to varying methodological rigor of published studies (e.g., poorly controlled studies with different instructors, or with no data provided on student or instructor equivalence).

Heterogeneity analyses also indicated no significant variation when comparing (a) studies with different research design such as historical control, quasi-experiment, or randomized experiment (*Q* = 0.52, df = 2, *p* = 0.77); (b) studies with different types of students such as medicine, pharmacy (*Q* = 2.44, df = 5, *p* = 0.78); (c) studies that employed pre-class assessment/exercise, or not (*Q* = 2.67, df = 1, *p* = 0.10); or (d) studies that provided pre-class readings/notes, or not (*Q* = 0.11, df = 1, *p* = 0.74).

However, a heterogeneity analysis in Table 3 indicated that the effect size is significantly higher when instructor(s) employed quizzes at the *start* of an in-class session to assess students’ learning of the pre-class video contents as opposed to instructor(s) who did not (*Q* = 5.34, df = 1, *p* = 0.02).

**Table 3:** Moderator analyses

Moderator				95% CI	
	*n*	SMD	SE	LL	UL
For student equivalence					
No data provided	13	0.31	0.10	0.11	0.50
No statistical difference (pretest, or other scores)	11	0.39	0.10	0.18	0.59
Randomized assignment	4	0.28	0.18	−0.08	0.63
For instructor equivalence					
No data	17	0.23	0.08	0.07	0.39
Different instructors	3	0.39	0.19	0.01	0.76
Identical instructors	8	0.55	0.12	0.31	0.79
Research design					
Historical control	20	0.32	0.08	0.17	0.48
Quasi-experiment	4	0.45	0.18	0.09	0.81
Random	4	0.28	0.18	−0.08	0.63
Types of students					
Medicine	13	0.26	0.10	0.08	0.45
Pharmacy	10	0.45	0.10	0.24	0.65
Public health	2	0.40	0.29	−0.18	0.98
Nursing	1	0.08	0.36	−0.62	0.79
Dental	1	0.19	0.30	−0.39	0.78
Chiropractic	1	0.29	0.39	−0.47	1.04
Availability of pre-class assessment/ exercise?					
No	17	0.25	0.08	0.09	0.41
Yes	11	0.46	0.10	0.26	0.65
Availability of pre-class readings/ notes?					
No	18	0.35	0.08	0.19	0.51
Yes	10	0.30	0.11	0.08	0.53
Availability of quiz at *start* of in-class?					
No	20	0.26	0.07	0.12	0.38
Yes	8	0.56	0.11	0.34	0.78*

*n* number of studies, *SMD* standardized mean difference, *SE* standard error, *95% CI* 95% confidence interval, *LL* lower limit, *UL* upper limit, *p < 0.05

### Publication bias

**Fig. 3:**
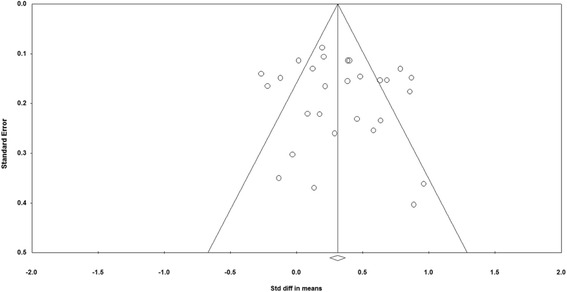
Funnel plot assessing publication bias

Visual inspection of Fig. 3 suggested no presence of publication bias. This is supported by two statistical analyses: Begg and Mazumdar rank correction (Kendall’s Tau with continuity correction) = 0.08, one-tailed *p* = 0.27; and Egger’s regression intercept 0.79, one-tailed *p* = 0.24. Computation of Duval and Tweedie’s trim and fill method using the random effects model revealed no studies were trimmed using the random effects model. We also conducted a classic fail-safe *N* test to determine the number of null effect studies needed to raise the *p* value associated with the mean effect above an arbitrary alpha level (α = 0.05). Results showed that 747 additional missing studies with zero mean effect size would be required to make the overall effect statistically insignificant. There would therefore have to be an unreasonably large number of undetected studies with zero effect to bring the effect sizes reported in this paper to values that might be statistically insignificant. Based on the visual inspection of funnel plot, statistical analyses, and class fail-safe *N*, we believe that the overall mean effect size is not inflated by publication bias.

### Analysis of learners’ preference

In the course of the review, we found five articles explicitly compared student preference for flipped or traditional classroom via survey questionnaires (Table 4). Overall, among the studies listed in Table 4, preference for flipped classroom was reported by an average of 70% of total respondents.

**Table 4:** Summary of survey results on student preference for flipped or traditional classroom

Study	Prefer flipped classroom *n* (%)	Prefer traditional classroom *n* (%)	Total respondents
Cotta et al. (2016) [13]	87 (73)	33 (27)	120
Galway et al. (2014) [34]	9 (82)	2 (18)	11
Giuliano and Moser (2016) [36]	49 (60)	33 (40)	82
^a^Kiviniemi (2014) [39]	33 (89)	4 (11)	37
Porcaro et al. (2016) [46]	50 (71)	20 (29)	70
*Total*	228 (71)	92 (29)	320

^a^The remaining 3 participants in Kiviniemi [57] indicated a preference for a combination of flipped and traditional classrooms

## Discussion

Overall, the data reported in this study indicate that more students favored the flipped classroom approach over traditional classroom. In addition, the flipped classroom approach was more effective than traditional classroom in increasing student learning performance. One explanation for the more positive student perception, as well as the greater effect of flipped classroom over traditional classroom, is that having unrestricted access to pre-recorded video lectures before class enables students to learn anywhere and at any time, at their own pace [36]. Students can also watch the videos multiple times to better understand a particular topic [13, 36]. Another explanation is the availability of more in-class active learning time to help increase students’ understanding of the subject material. Many of the in-class activities such as small-group discussion promoted students’ interactions with their peers [34]. Instructors also felt they had greater opportunity to provide more feedback during in-class sessions [34]. There were also greater opportunities for students to apply their knowledge in flipped classes [13, 34].

Further analyses suggest that the increase in performance holds across studies of different research designs or methodological quality. Experiments where students were randomly assigned to flipped classrooms produced results that were indistinguishable from quasi-experiments and historical controls. Analyzing variation with respect to controls over student or instructor equivalence also produced no evidence of heterogeneity. In addition, the availability of online assessment/exercises, or readings *before* face-to-face class did not appear to moderate performance gains, as no heterogeneity was detected between the subgroups.

However, we found that the use of quizzes at the start of a face-to-face class would make flipped classroom more effective. The quizzes consisted of specific questions that were developed by the instructor beforehand and were used to assess student learning of the pre-class learning materials. One explanation for this finding is that quizzes at the beginning of class helped students recall the knowledge learned prior to the class. Prior knowledge has long been considered an important factor influencing learning [54, 55]. Stimulating the recall of prior knowledge helps learners to make better sense of new information by connecting it to something they already know. In addition, the retrieval of information from memory makes the path to that information in memory stronger; this consequently enables the information to be more easily retrieved by the learner on the next occasion [56].

Having quizzes at the beginning of class also allows an instructor to identify students’ possible misconceptions of the pre-class materials. Students’ misconceptions can prevent further learning if not addressed. Based on student performance, instructors can provide remedial action if necessary such as reviewing the pre-class video lectures or making adjustments to the in-class teaching plans to specifically address the students’ misconceptions. The use of quizzes at the beginning of an in-class lesson can also serve as a strong motivator for students to watch the pre-class video lectures [34, 50]. This finding thus implies that instructors use quizzes as a regular part of the in-class activities to assess students’ mastery of the pre-class learning materials.

Students who preferred a traditional classroom reported that watching video lectures took a lot of additional time [13]. In a traditional class, students learn about the subject matter through a teacher-led lecture format during class time [13]; however in a flipped class students are now required to watch the video lectures before class. Students were unhappy being asked to do work at home that was traditionally done in a face-to-face class format, and considered watching the pre-class videos as burdensome in terms of time [13]. Studies from several non-health professions education flipped classrooms also supported this finding. For example, half of the students who would unwilling to take another flipped class cited the additional time required to complete the pre-class work as a reason [57]. This finding thus implies that instructors who wish to employ flipped classroom should first promote students’ understanding of this new instructional approach by explaining the rationale, and potential benefits of flipped classroom [20]. In addition, instructors may consider limiting total length of all *combined* video segments to about 20 min. Support for this comes from several non health professions education-related flipped classroom studies [58, 59] which reported that most students spent up to 20–25 min on viewing pre-class video lectures.

### Strengths and limitations

Our meta-analysis has several strengths. The flipped classroom approach has grown rapidly and is now widely used in health professions education. To our knowledge, this is the first meta-analysis to summarize the evidence to date concerning the effectiveness of flipped classroom on student learning compared with traditional classroom. Evaluating the effectiveness of flipped classroom is therefore both timely and important for instructors and learners. We also intentionally kept our literature search very recent (up to April 15, 2017) and broad in terms of subjects from various health professions using multiple academic online databases.

However, there are some limitations that should be considered. First, this review focused on flipped classroom studies in which pre-class videos were provided, and class attendance was mandatory. To broaden the scope of review, future reviews can examine other flipped learning studies that do not restrain the instructors’ use of technological tools. Future reviews can also compare the use of online course without face-to-face meeting and flipped classes with face-to-face meeting. Second, the flipped classroom designs in the reviewed studies were not always clearly reported. For example, the specific types of video lecture used were not described. Video lectures can include many different styles including recorded classroom lecture, Khan-style freehand writing video, PowerPoint presentation with instructor talking head, PowerPoint presentation with more than one people in conversation, among others [60–62]. Different video styles may affect student learning. We also could not identify information related to the actual time allocated to different instructional activities (e.g., small-group activities), and the actual details of small-group learning activities. Specifically, with regard to the small-group learning activities, there is no consensus about what various activities actually entail in practice [27]. As a result, we could not really differentiate the activities when the authors merely stated the use of small-group discussion, in-class collaboration, or group problem solving without providing specific details on the actual tasks involved in the group activities because group discussion, collaboration, problem-solving all involved discussions; and problem-solving can also be a form of collaboration. The absence of all this information *prevented* us from conducting further moderator analyses to discern *additional key factors* that could affect flipped classroom effectiveness. Additionally, the results are limited because no study included long-term follow-ups to assess learning retention. Also, the overall effect size for randomized controlled trials is usually bigger than for cohort studies.

## Conclusions

Current evidence suggests that the flipped classroom approach in health professions education overall yields a statistically significant improvement in learner performance compared with traditional teaching methods. In addition, the flipped classroom would be more effective when instructors use quizzes at the start of each in-class session. Future research can be conducted to examine the possible effect of specific types of teaching method or presentation on student learning. Future research should also examine the possible impact of video styles. Despite the increasing popularity of using video-recorded lectures, we still understand little about how different video styles may impact student learning. Longitudinal studies should also be conducted to examine whether the flipped classroom approach can foster learning retention over a long period of time.

## Abbreviations

CI: Confidence interval
EBM: Evidence-based medicine
LL: Lower limit
MERSQI: Medical education research study quality instrument
PRISMA: Preferred reporting items for systematic reviews and meta-analysis
SE: Standard error
SMD: Standardized mean differences
TED: Technology, entertainment, design
UL: Upper limit
